# No Genetic Tradeoffs between Hygienic Behaviour and Individual Innate Immunity in the Honey Bee, *Apis mellifera*

**DOI:** 10.1371/journal.pone.0104214

**Published:** 2014-08-27

**Authors:** Brock A. Harpur, Anna Chernyshova, Arash Soltani, Nadejda Tsvetkov, Mohammad Mahjoorighasrodashti, Zhixing Xu, Amro Zayed

**Affiliations:** Department of Biology, York University, Toronto, Ontario, Canada; University of North Carolina, Greensboro, United States of America

## Abstract

Many animals have individual and social mechanisms for combating pathogens. Animals may exhibit short-term physiological tradeoffs between social and individual immunity because the latter is often energetically costly. Genetic tradeoffs between these two traits can also occur if mutations that enhance social immunity diminish individual immunity, or vice versa. Physiological tradeoffs between individual and social immunity have been previously documented in insects, but there has been no study of genetic tradeoffs involving these traits. There is strong evidence that some genes influence both innate immunity and behaviour in social insects – a prerequisite for genetic tradeoffs. Quantifying genetic tradeoffs is critical for understanding the evolution of immunity in social insects and for devising effective strategies for breeding disease-resistant pollinator populations. We conducted two experiments to test the hypothesis of a genetic tradeoff between social and individual immunity in the honey bee, *Apis mellifera*. First, we estimated the relative contribution of genetics to individual variation in innate immunity of honey bee workers, as only heritable traits can experience genetic tradeoffs. Second, we examined if worker bees with hygienic sisters have reduced individual innate immune response. We genotyped several hundred workers from two colonies and found that patriline genotype does not significantly influence the antimicrobial activity of a worker’s hemolymph. Further, we did not find a negative correlation between hygienic behaviour and the average antimicrobial activity of a worker’s hemolymph across 30 honey bee colonies. Taken together, our work indicates no genetic tradeoffs between hygienic behaviour and innate immunity in honey bees. Our work suggests that using artificial selection to increase hygienic behaviour of honey bee colonies is not expected to concurrently compromise individual innate immunity of worker bees.

## Introduction

Organisms can individually fight pathogens by mounting an immune response that ultimately results in the synthesis and release of antipathogenic molecules that kill or inhibit the growth of pathogens. Moreover, organisms that regularly interact with conspecifics can engage in social behaviors that reduce the loads and transmission rates of pathogens (i.e. social immunity) [1]. For example, the social ants, bees and wasps, have an impressive array of social immune behaviours, which include grooming, social fever, exclusion of infected individuals, and hygienic behaviour [2], [3]. Mounting an individual innate immune response is energetically costly, and this cost is hypothesized to result in tradeoffs between innate immunity and other traits important to survival and reproduction over short (i.e. physiological) and long (i.e. evolutionary) timescales [4].

Physiological tradeoffs between innate and social immunity have been discovered in the sub-social burying beetle *Nicrophorus vespilloides*, who rear their brood on carrion. In this species, adults synthesize antimicrobial enzymes that can be used for personal immunity or for ‘sanitizing’ the carcass used for brood rearing (i.e. social immunity). The physiological cost of synthesizing antimicrobials in this species leads to a tradeoff between investment in personal immunity or social immunity over short-timescales [5]. Several researchers have also documented physiological tradeoffs between innate immunity and cognitive functions that may be important for social immunity in insects. For example, Mallon et al. [6] challenged the immune system of honey bees with a non-pathogenic elicitor and found that the resulting innate immune response reduced the bees’ ability for associative learning. In other words, bees mounting an immune response learn poorly. Similar findings were reported for social bumble bees [7], [8]. This short-term tradeoff between innate immunity and learning may impede social immunity if bees have to learn odor cues of sick sisters for example.

Tradeoffs between two traits can also occur for genetic reasons. A genetic tradeoff occurs when mutations influence two traits in an antagonistic manner (i.e. antagonistic pleiotropy) [9], [10], by enhancing one trait and diminishing another. Genetic tradeoffs are uncovered by demonstrating negative associations between two traits among groups of related individuals [11]. For example, Alghamdi et al. [11] studied the potential for a genetic tradeoff between innate immunity and learning by examining if bumblebees with fast-learning sisters have reduced innate immunity; the authors did not observe this pattern. In other words, healthy bees with fast-learning genotypes do not have reduced innate immunity.

Although there is some evidence for a physiological tradeoff between social and innate immunity, we know little about the existence of genetic tradeoffs between these two components of the immune system of social insects. Several molecular studies of the honey bee *Apis mellifera* have proven the existence of genes with pleiotropic effects on worker behaviour and innate immunity. For example, Chandrasekaran et al. [12] discovered that the honey bee ortholog of *NF-kB*, is a major transcription factor that globally regulates several thousand genes associated with behaviour of honey bee workers. *NF-kB* is also a key regulator of innate immunity in honey bees and solitary insects [13], [14]. Further, several genes that are differentially regulated in the brains of worker bees during behavioural maturation also play a molecular role in innate immunity [15]. The presence of pleiotropic genes that influence innate immunity and worker behaviour in honey bees suggests the potential for genetic tradeoffs between these traits in social insects.

Understanding the genetic relationship between social and innate immunity is critical for understanding the evolution of immunity in social insects. This knowledge is needed to better understand why some social insects, like the honey bee, have a reduced complement of innate immune genes [14], [16]. Understanding the genetic relationship between innate and social immunity is also critical for developing effective strategies for managing the health of natural and managed pollinators [17], [18]. For example, artificial selection for social immunity is commonly used in the management of honey bee colonies in Europe and North America [19], [20], but this strategy may actually decrease innate immunity if there is a genetic tradeoff between these two traits in honey bees.

We conducted two experiments to study genetic tradeoffs between innate immunity and hygienic behaviour – an important component of social immunity in honey bees [3]. Negative genetic correlations (i.e. tradeoffs) between hygienic behaviour and innate immunity can only occur if both traits are heritable [21]. There is overwhelming evidence that hygienic behaviour has high heritability in honey bees [22], [23]. There is also evidence of a genetic contribution to variation in disease resistance in honey bee colonies [24]–[26], and one study found the expression of an antimicrobial peptide, abaecin, to be heritable in honey bees [27]. However, we lack information on the heritability of innate immune function on a broader scale in honey bees. For the first experiment, we genotyped 309 workers from two colonies to determine the broad-sense heritability of the antimicrobial activity of a worker’s hemolymph, using a zone of inhibition assay following an immune challenge [28]–[30]; this assay was used to demonstrate heritability of innate immune response, and map quantitative trait loci affecting this response in a bumble bee [31]. Second, we sought to determine if honey bees from hygienic colonies have low individual immunity, and vice versa. To do so, we quantified individual innate immunity and hygienic behaviour across 30 typical managed honey bee colonies. Our study sheds light on the relationship between innate and social immunity in a model eusocial insect, and has important ramifications for using artificial selection to improve the health of managed pollinators.

## Materials and Methods

### Quantifying innate immune response

To measure the antimicrobial activity of a worker’s hemolymph, we utilized the zone of inhibition assay (ZOI) following injection of lipopolysaccharide (LPS). LPS is a non-pathogenic elicitor of the innate immune system; injecting workers with LPS elicits an immune response leading to the release of anti-pathogenic effector proteins into the hemolymph [29], [32]. The ZOI assay has been widely used in the study of innate immunity of social insects [28]–[31]. We performed the assay by first chilling and lightly restraining worker bees (1-day olds for experiment 1, hive bees for experiment 2; see justification below) and sterilizing their abdomen with a Kimwipe saturated in 70% ethanol. Bees were then injected between the 2^nd^ and 3^rd^ tergite with 1.5 µl of 0.5 mg/ml LPS dissolved in sterile insect saline solution (128 mM NaCl, 18 mM CaCl_2_, 1.3 mM KCl, 2.3 mM NaHCO_3_, pH 7.2) [30]. The bees were then placed in cages and fed sugar water (2∶1) *ad libidum* in a laboratory incubator set to 33°C. Twenty-four hours after LPS injection, bees were again chilled and restrained, and at least 4 ul of hemolymph was collected per bee onto dry-ice [30]. To measure the antibacterial activity of the hemolymph 24 hours after LPS injection, we pipetted 1 ul of hemolymph onto a bacterial plate containing a lawn of *Arthrobacter globiformis*. We grew a liquid bacterial stock using 5 µl of concentrated frozen stock (1.6×10^9^ cells per ml) mixed in 50 ml of sterilized broth solution, and incubated at 30°C for 48 hrs [30]. From this stock, 1 ml was used to inoculate 5 ml agar plates. The agar on each plate was also punctured with 10 evenly spaced 2 mm holes to which hemolymph was added before being incubated again at 30°C for 48 hrs.

To control for plate-to-plate variation in bacteria density, we also added 1 µl of 1∶200 (high dose) and 1∶300 (low dose) dilutions of dihydrostreptomycin antibiotic (0.1 g/ml) in 80% glycerol on each plate. The ZOI produced by the two antibiotic controls were highly correlated (p < 5.3×10^−−13^, r = 0.75). The plate was incubated at 28°C for 24 hrs, after which the diameter of each zone of inhibition of each sample was measured in triplicate. We standardized the area of the zone of inhibition for each experimental sample by dividing it by the area of the zone of inhibition generated by the high dose of the antibiotic control on each plate. As such, all ZOI measurements are presented as percentage of the antibiotic control.

We performed a series of experiments to validate that LPS injection is eliciting an immune response in worker bees. We compared the ZOI scores of nurse bees that were sham-handled (e.g. chilled and restrained, but not injected; n = 8 bees, ZOI: 0.74 mm +/− 0.266 SE), injected with only saline solution (n = 10, ZOI: 1.36 mm +/− 0.17 SE), and LPS + Saline injected bees (n = 8, ZOI: 2.22 mm +− 0.24 SE) (Dataset S1). We found that the treatments had a significant effect on ZOI scores (ANOVA: F = 10.14, df = 2, 23, p < 0.001). The ZOI scores of LPS-injected bees were significantly higher relative to sham-handled (p < 0.001) or saline-injected bees (p < 0.05), but the ZOI scores of sham-handled bees and saline-injected bees did not significantly differ (p = 0.141). Our analysis validates that LPS injections elicit an immune response in honey bee workers.

### Experiment 1: Broad-sense heritability of the antimicrobial activity of a worker’s hemolymph

Queen honey bees mate with a large number of males (usually 15 to 20), which results in a large number of patrilines within a colony [33]. Because of male-haploidy, workers sired by the same father are 75% related, while workers sired by different fathers are 25% related. All the patrilines are reared in the same environment and experience the same maternal effects, allowing us to directly quantify the relative contribution of genetic variance to phenotypic variance [34]–[37]. In this context, broad-sense heritability of a phenotype (*H* ^2^) can be estimated by contrasting the phenotypic variance that is attributed to patriline identity relative to the total phenotypic variance within a colony using analysis of variance [34], [37]; *H* ^2^ is twice the patriline variance divided by the total phenotypic variance.

We collected frames of brood from two honey bee colonies (c2 and c10) maintained at York University’s Research Apiary (Toronto, Canada) in the summer of 2012. The managed bees have mixed genetic ancestry with major contributions from the East Europe population group (C group: *A. m. ligustica* and *A. m. carnica*) and minor contributions from the West Europe population group (M group: *A. m. mellifera*) [38], [39]. Brood frames were incubated at 33°C, and checked daily for emerging bees. Over a period of several weeks, we quantified the antimicrobial activity of hemolymph extracted from these 1-day-old workers using the ZOI assay described above. 1-day-old workers treated with LPS exhibit a strong inducible immune response relative to non-injected, and sham-injected controls [30]. We studied 1-day-old workers for experiment 1 to eliminate the confounding influence of age and behavioural state on worker physiology and gene expression [40]. To eliminate potential confounds from infection status, only workers that appeared healthy were used in this study; workers with deformed wings or phoretic *Varroa* mites were excluded. After the ZOI assay, workers were immediately frozen on dry ice and left at −80°C until genetic analysis.

We genotyped each worker at 9 microsatellite loci to determine patrilines. DNA was extracted from one hind leg per bee using Chelex following established protocols [41]. We amplified the following hyper-variable microsatellite loci, shown previously to be useful in patriline studies of honey bees [42]: HB-SEX-02, HB-THE-03, AC006, HB-C16-05, HB-SEX-03, HB-C16-01, A024, A107, and A007. We amplified these 9 loci in two multiplex reactions following a published protocol [42]. The reactions were sent to Genome Québec Innovation Centre at McGill University for automated fragment analysis. We used Genemapper (version 4.0) to designate alleles and call genotypes, which were checked manually for errors and miscalls. We were able to genotype 8 loci (all except A107) in colony c10, and 7 loci (all except A107 and A007) in colony c2. To distinguish patrilines, we first deduced the genotype of the mother queen using the genotypes of her daughters; a heterozygous mother will pass on two alleles at equal frequency to her daughters, while a homozygous mother will pass on a single allele to all of her daughters. After deducing the maternal allele, we estimated the paternal allele at a locus in a worker by subtraction. We assumed that two or more workers with the same paternal haplotype were sired by the same father. We excluded rare patrilines (i.e. with 3 or fewer daughters) because these could arise from genotyping error (16 and 22 workers were excluded from c10 and c2 respectively using this criteria). We used general linear models to determine if colony and patriline have a significant effect on the antimicrobial activity of a worker’s hemolymph using the GLM procedure in SAS Ver. 4. We also conducted nested analysis of variance to estimate the variance component associated with patriline and colony, using the NESTED procedure in SAS. Post-hoc pairwise comparisons between patrilines were conducted using Tukey’s HSD function in R version 3.0.1 [43].

### Experiment 2: Phenotypic correlation between innate immunity and hygienic behaviour

Workers within a colony have substantial levels of genetic relatedness (e.g. 75% among full sisters, 25% among half sisters). We would predict workers from colonies that have hygienic sisters, due to the presence of common genetic variants influencing this trait within a colony, to have relatively low average individual immunity, assuming a genetic tradeoff between the two traits. We studied 30 managed colonies at York University’s Research Apiary during the summer of 2012. These included ten one-year-old 2-storey Langstroth colonies each with 20 deep frames and twenty 2 to 3 month-old 4-frame colonies. Both young and old colonies were headed by naturally mated queens, and were chosen to have similar population sizes prior to the experiment (i.e. old colonies were of similar size relative to other old colonies, and young colonies were of similar size relative to young colonies). For each colony, we quantified hygienic behaviour using a ‘freeze kill’ assay, which involves using liquid nitrogen to freeze a section of capped brood cells, followed by measuring the amount of dead brood removed by workers 24 hours later [44]–[47]. We took before and after pictures, and quantified hygienic behaviour as the percentage of frozen brood removed by workers within 24 hours [44].

We also quantified the relative amount of mites (*Varroa destructor*) in each colony using the “sugar shake” method [48]. We placed 250 mL of hive bees (i.e. bees on frames containing brood) into glass jars with 125 mL of fine icing sugar [48]. The jars were shaken until all the bees were coated in sugar; this dislodges phoretic mites into the powdered sugar remaining in the jar [48]. The sugar was collected from the jars, dissolved in hot water, and poured through a fine mesh to trap mites. The number of mites per ml of bees was used for analysis.

From each colony, we also collected 10 hive bees and assessed the antimicrobial activity of their hemolymph using the ZOI assay; as with experiment 1, only workers that appeared healthy were used in this study; workers with deformed wings or phoretic *Varroa* mites were excluded. The average of the antimicrobial activity from each colony was used to represent the innate immune capacity of the colony. We then conducted linear correlations between colony innate immunity and colony hygienic behaviour using R version 3.0.1 [43]. We studied hive bees for Experiment 2 because hive bees (i.e. bees performing in-hive activities and not foraging) encompass a subset of workers that perform hygienic behaviour (i.e. hive bees which range in age from 8 to 22 days perform hygienic behaviour [46], [47]). Hive bees are also more susceptible to infection, and mount a stronger innate immune response relative to foraging bees [49], [50]. We did not sample hive bees that were actively engaged in hygienic behaviour for the ZOI analysis because this would have potentially confounded physiological tradeoffs with genetic tradeoffs [8], [11], if both existed. Although it would have been ideal to collect bees of known age for experiment 2, it was not practical given that the ZOI assay requires live bees and is difficult to perform on hundreds of workers of the same age at the same time. We collected hive bees of unknown age from all colonies in a consistent manner, and the average age of hive bees in hygienic colonies is not expected to differ across our study colonies.

## Results

### Experiment 1: Broad-sense heritability of innate immune response

**Figure 1:**
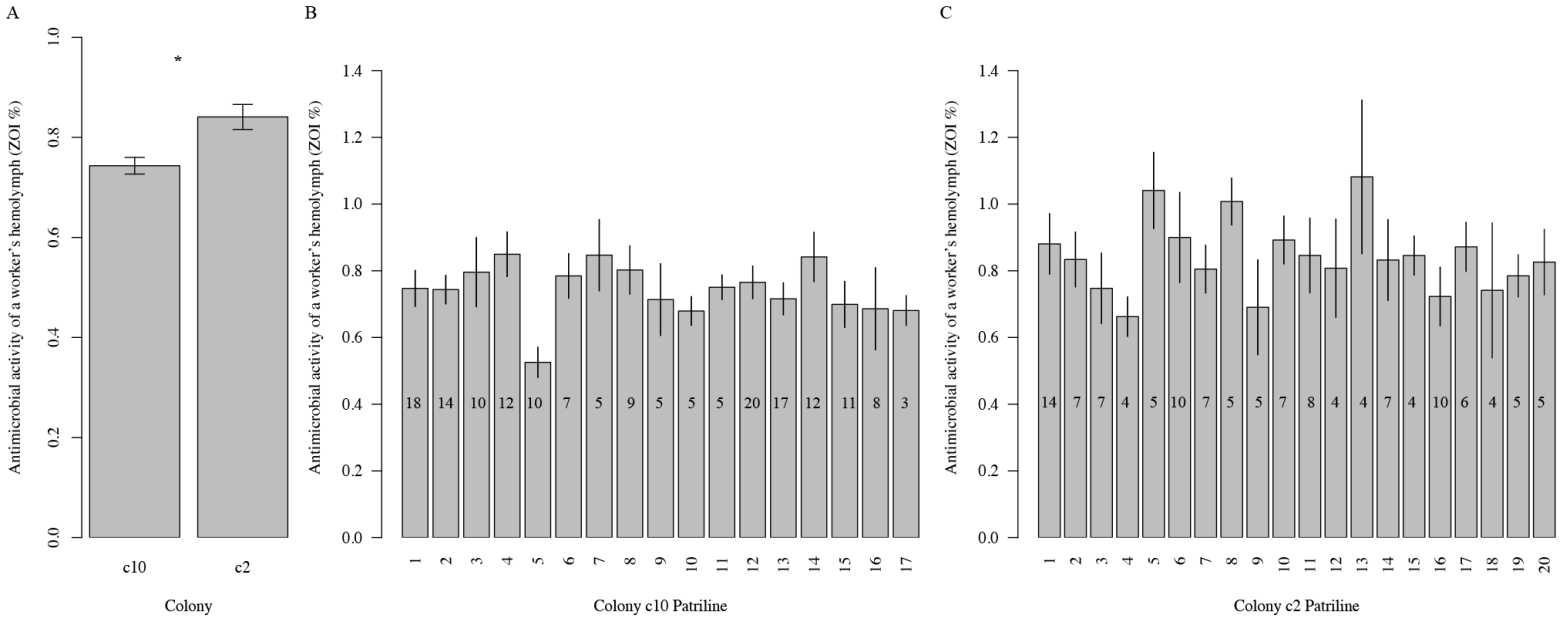
The antimicrobial activity of a worker’s hemolymph differs across colonies but not across patrilines. (A) The average antimicrobial activity of a workers’ hemolymph is significantly larger in colony c2 than colony c10. Workers from different patrilines did not significantly differ with respect to their average antimicrobial activity of their hemolymph in (B) colony c10 and (C) colony c2. Error bars indicate standard error of the mean. Sample size of each patriline is indicated on each bar.

We quantified the strength of innate immune response and patriline identity for a total of 309 workers belonging to two colonies (c2 and c10). Colony c2 and c10 contained a total of 20 and 17 patrilines; well within the range for colonies headed by naturally mated queens [35], [36]. Patrilines in colony c10 ranged in size from 5 to 20 workers, with an average of 10.6 workers. Patriline in colony c2 ranged in size form 4 to 14 workers, with an average of 6.4 workers. Workers from colony c2 had significantly higher hemolymph antimicrobial activity (0.84 +/− 0.25 SE) relative to workers from colony c10 (0.74 +/− 0.16 SE; ANOVA, F = 11.41, df = 1, p = 0.0018; Figure 1A, Dataset S2). Workers within both colonies exhibited a great degree of variation in the antimicrobial activity of their hemolymph (figure 1). We found no significant effect of patriline identity on the antimicrobial activity of a worker’s hemolymph in colony c10 (F = 1.28, df = 16, p = 0.22; Figure 1B) and c2 (F = 0.73, df = 19, p = 0.78; Figure 1C). Using a generalized linear model with colony as a factor, and patriline identity as a nested factor (i.e. nested within colony), we detected a significant effect of colony (df = 1, p = 0.002), but not patriline identity (df = 35, p = 0.49) on the antimicrobial activity of a worker’s hemolymph. We conducted a nested random effects analysis of variance to partition the total variance in the antimicrobial activity of a worker’s hemolymph to the following components: 93.5% among workers; 0% among patrilines; and 6.5% among colonies. A similar pattern emerged when each colony was analyzed separately; the among-patrilines variance component is 2.56% in colony c10, and 0% in colony c2. We repeated the analyses above but only included large patrilines (see Table S1 for different cutoffs); all such analyses confirmed our finding that patriline identity has a small and statistically insignificant effect on the antimicrobial activity of a worker’s hemolymph.

Although patriline identity was not a significant factor influencing the antimicrobial activity of a worker’s hemolymph across our experiment, we performed post-hoc tests to determine if workers from any two pairs of patrilines exhibit differences in the antimicrobial activity of their hemolymph. In colony c2, all pairwise comparisons of the average antimicrobial activity of a worker’s hemolymph between patrilines were not significant (Tukey HSD, p > 0.85 for all tests). A similar pattern was observed in colony c10, although patriline 5 was marginally lower than patriline 4 (Tukey HSD, p = 0.064) and patriline 14 (p = 0.082). Our results suggest that genetic differences are not responsible for the majority of the differences in the antimicrobial activity of a worker’s hemolymph in our studied colonies.

### Experiment 2: Phenotypic correlation between innate immunity and hygienic behaviour

**Figure 2:**
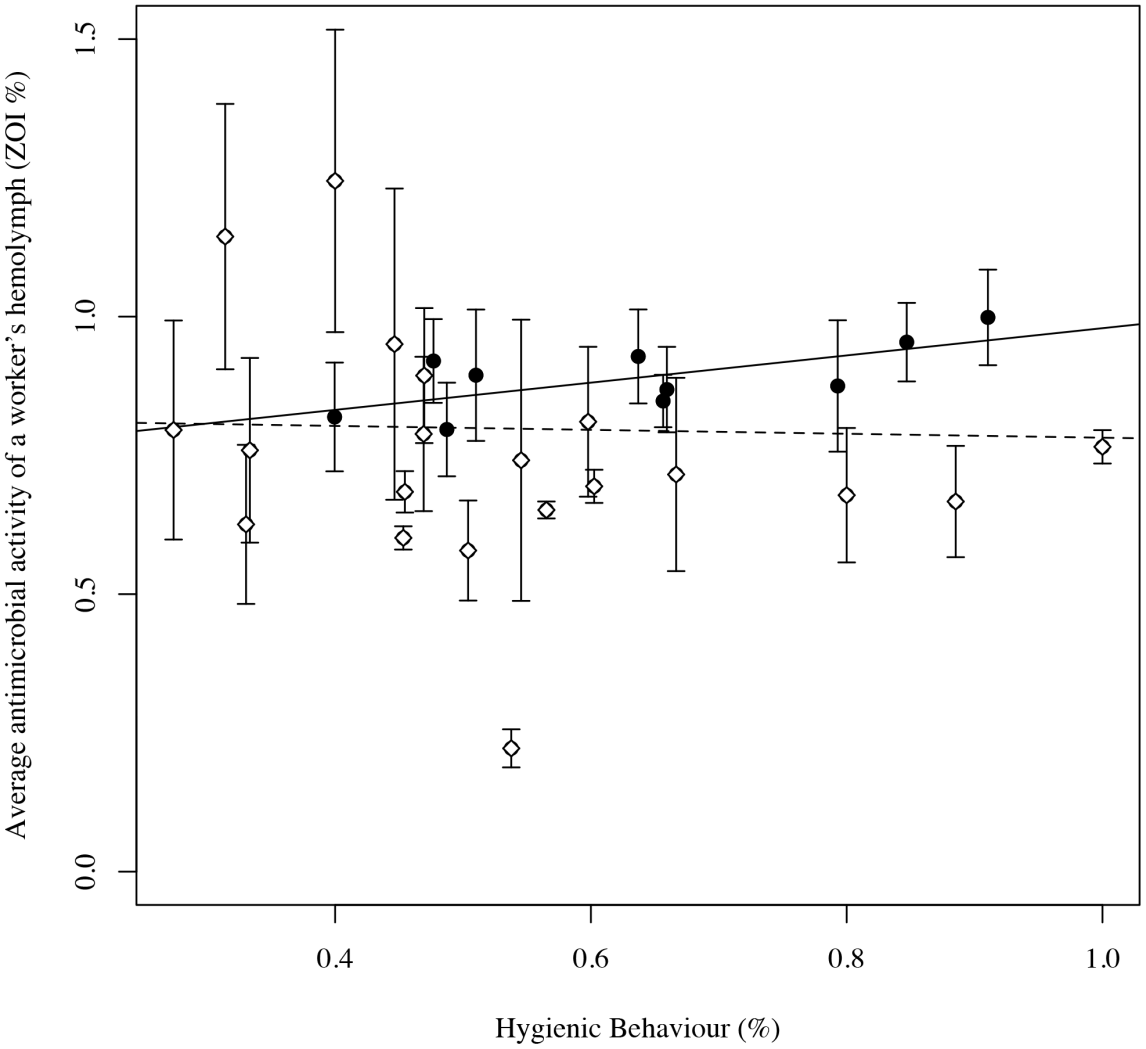
The average antimicrobial activity of a workers’ hemolymph is not significantly correlated with hygienic behaviour across 30 managed honey bee colonies (dashed line). Among old colonies (>1 year old, black circles), we found a significant positive correlation between individual innate immunity and hygienic behaviour (black line). Error bars indicate standard error of the mean.

We quantified hygienic behaviour and the average antimicrobial activity of a worker’s hemolymph from 30 colonies in our research apiary; ten colonies were established for 1 year or longer (henceforth old), while the others were 2 to 3 months old (henceforth young) (Dataset S3). Across all colonies, we found no significant correlation between hygienic behaviour and the average antimicrobial activity of a worker’s hemolymph (Pearson, t = −0.1886, df = 28, r = −0.04, p-value = 0.85; Figure 2). However, we observed that older colonies tended to have higher levels of hygienic behaviour (ANOVA, F = 2.1798, df = 1, p = 0.15) and higher hemolymph antimicrobial activity (F = 4.1437, df = 1, p = 0.051), and the latter difference approached statistical significance. Older and younger colonies did not differ with respect to *Varroa* mite levels (ANOVA, F = 0.254, df = 1, p = 0.6181). When analyzed separately, older colonies exhibited a significant positive correlation between hygienic behaviour and the average antimicrobial activity of a worker’s hemolymph (Pearson, t = 2.6218, df = 8, r = 0.68, p-value = 0.03). In young colonies, we found no significant relationship between hygienic behaviour and the average antimicrobial activity of a worker’s hemolymph (t = −1.1513, df = 18, r = −0.26, p-value = 0.26). Both of these patterns are inconsistent with the hypothesis of a genetic tradeoff inducing a negative correlation between the two traits [11].

## Discussion

Our study provides two lines of evidence against genetic tradeoffs between social and innate immunity in the honey bee. First, genetic tradeoffs between two traits can only occur if both are heritable, and if genetic variants that influence one trait have antagonistic effects on the other [21]. The results of experiment 1 preclude the possibility of a genetic tradeoff between the antimicrobial activity of a worker’s hemolymph and the hygienic behaviour of a colony because the antimicrobial activity of a worker’s hemolymph had very low and statistically insignificant broad-sense heritability (*H^2^∼0*) in our study. It is important to note that this low heritability is unlikely to be caused by low statistical power, for even if we ignore that patriline identity was not a significant factor in the study, or if we restricted our analyses to large patrilines, the variance component associated with this factor was still minuscule. Second, we found no negative phenotypic correlation between the average antimicrobial activity of a worker’s hemolymph and hygienic behaviour across 30 typical honey bee colonies. In fact, among older colonies, we observed a significant positive phenotypic correlation between innate immunity and hygienic behaviour; the opposite of what would be predicted from a tradeoff. It is difficult to speculate why older colonies would show a positive relationship between hygienic behaviour and innate immunity, while younger colonies do not. Younger colonies are smaller than older colonies, and small colonies tend to have lower hygienic behaviour relative to large colonies [51].

A recent study by Wilson-Rich et al. [52] found that patriline genetics does not influence two aspects of innate immunity in honey bees: encapsulation response and the phenoloxidase activity of a worker’s hemolymph. Our study extends Wilson-Rich et al.’s findings by showing that patriline does not substantially influence the antimicrobial activity of a worker’s hemolymph. Previous work by Decanini et al. [27] found the expression level of the antimicrobial peptide abaecin to be heritable in honey bees. Our finding of low and insignificant broad sense heritability for the antimicrobial activity of a worker’s hemolymph is not entirely at odds with Decanini et al.’s study because the ZOI assay quantifies the strength of innate immunity at a very broad scale. Many proteins contribute to the antimicrobial activity of the hemolymph [53] and heritability of expression of a single gene is unlikely to drive heritability of the antimicrobial activity of a worker’s hemolymph. We believe that the ZOI assay provides an appropriate high-level measure of innate immune response, and previous authors have demonstrated its utility in quantitative genetic studies of innate immunity in social insects [31].

### Can an evolutionary tradeoff exist without a genetic tradeoff?

We found no genetic tradeoffs between hygienic behaviour and individual immunity in honey bees, which – at first glance – appears at odds with several hypothesis invoking an evolutionary tradeoff between individual and social immunity in this species [14], [16], [54]. Before addressing this important question, we would first like to clarify some definitions because genetic and evolutionary tradeoffs are often used as synonyms [11], [16]. Here we define an evolutionary tradeoff as a gain or elaboration of a trait that is correlated with the loss or reduction of another trait over evolutionary timescales (e.g. elaboration of social immunity and reduction in innate immune genes in honey bees). We use the standard quantitative genetic definition of a genetic tradeoff as a negative genetic covariance between two traits due to pleiotropy [21]. Given these definitions, a genetic tradeoff can lead to an evolutionary tradeoff between two traits assuming that one trait is under directional selection. However, an evolutionary tradeoff may occur without a genetic tradeoff, so long as selection pressures favor evolution of one trait relative to another.

If social immunity is physiologically less costly than innate immunity [5], [14], [16], and if this physiological cost is not transient (i.e. lasts during the lifetime of an individual), then we would predict stronger positive selection on genes associated with social immunity and a concurrent relaxation of purifying selection on genes associated with innate immunity. Over evolutionary timescales, these different selection regimes should result in higher rates of adaptive phenotypic evolution of social immunity, and an evolutionary decay of superfluous innate immune genes. The honey bee has elaborate social immune defenses, and we’ve previously documented high rates of adaptive evolution of genes associated with worker behaviour [55], [56]. We have also shown that many innate immune genes in the honey bee have signatures of relaxed purifying selection [57]. Our independent population genetic evidence therefore supports the hypothesis of relaxed purifying selection on innate immune genes combined with concurrent positive selection on genes associated with worker behaviour. Given the importance of worker behaviour to colony fitness in honey bees [55], we speculate that physiological tradeoffs [6]–[8], but not genetic tradeoffs, between innate immunity and cognitive function, result in the elaboration of social immunity and the decay of innate immunity in honey bees over evolutionary timescales.

In addition to shedding light on the evolution of individual and group immunity in social insects, our findings are relevant for breeding disease-resistant honey bee colonies. Currently, artificial selection of colonies with hygienic behaviour is an effective tool for increasing the health of managed honey bee colonies [45], [46], [58]–[60]. If genetic tradeoffs existed between social and innate immunity, then selection for hygienic behaviour will ultimately compromise the individual innate immunity of worker bees, and may not improve the health of bee colonies on the whole. However, our findings reject the hypothesis of a genetic tradeoff between innate immunity and hygienic behaviour in honey bees. As such, selection for hygienic behaviour is not expected to influence individual innate immunity in managed colonies.

## Supporting Information

Table S1 Summary of different statistical analyses examining the influence of patriline identity on worker ZOI scores using patrilines with different size cutoffs. The table contains statistics from GLM and nested analysis of variance as described in the methods.
(DOCX)

Dataset S1 Validating the ZOI assay on hive bees. CON = bees chilled and restrained, but not injected. SAL = bees chilled, restrained, and injected with saline solution (i.e. sham-injected), LPS = bees chilled, restrained, and injected with saline solution + LPS.
(XLSX)

Dataset S2 ZOI scores for worker bees from colony c2 and c10. The patriline of each worker is indicated.
(XLSX)

Dataset S3 Mean and SE of ZOI scores for 10 hives bees, hygienic behaviour and mite loads for 30 colonies studied for experiment 2. Age: y = young colonies; o = old colonies; see manuscript for details.
(XLSX)
