# Supplementary material for: No Genetic Tradeoffs between Hygienic Behaviour and Individual Innate Immunity in the Honey Bee, Apis mellifera
Source: PLoS One. 2014 Aug 27;9(8):e104214. doi: 10.1371/journal.pone.0104214 (PMC4146461; doi:10.1371/journal.pone.0104214)
Supplement: Table S1 — Summary of different statistical analyses examining the influence of patriline identity on worker ZOI scores using patrilines with different size cutoffs. The table contains statistics from GLM and nested analysis of variance as described in the methods. (DOCX) [file pone.0104214.s001.docx]

|  | Patriline effect on ZOI scores | | | |
| --- | --- | --- | --- | --- |
| Patriline Cut-off  ≥ # worker | f | df | p | Variance component (%) |
| 14 | 0.14 | 3 | 0.9375 | 0 |
| 10 | 1.51 | 11 | 0.1313 | 3.57 |
| 5 | 0.91 | 21 | 0.5804 | 0 |
| 3 | 0.99 | 35 | 0.4955 | 0 |

Table S1: Summary of different statistical analyses examining the influence of patriline identity on worker ZOI scores using patrilines with different size cutoffs. The table contains statistics from GLM and nested analysis of variance as described in the methods.
